# The SHARP Exam: A Standardized POCUS Approach to Undifferentiated Acute Right Lower Quadrant Abdominal Pain in Young Women

**DOI:** 10.24908/pocusj.v10i02.18493

**Published:** 2025-11-17

**Authors:** Michael Halperin, Maia Winkel, Ashley Aiken, Nora McNulty, Michelle Montenegro, Nicole Leonard Shiu, Trevor Dixon, Alyssia McEwan, William Murk, Ariella Gartenberg

**Affiliations:** 1 Department of Emergency Medicine, Jacobi Medical Center and Albert Einstein College of Medicine, Bronx, NY, USA; 2 Department of Emergency Medicine, Stanford University, Palo Alto, CA, USA; 3 Department of Emergency Medicine, Baylor College of Medicine, Houston, TX, USA; 4 Department of Emergency Medicine, Vanderbilt University, Nashville, TN, USA

**Keywords:** Point of Care Ultrasound, Abdominal Pain, Right Lower Quadrant, Pregnancy, POCUS

## Abstract

Acute right lower quadrant (RLQ) pain is a common presenting complaint in emergency departments among young women. The SHARP Exam is a novel point of care ultrasound (POCUS) protocol designed to aid in the evaluation of undifferentiated, acute, right-sided, lower abdominal pain in women of child-bearing age. The SHARP Exam is both an acronym ([**S**]ono [**H**]er [**A**]bdomen for [**R**]ight-sided [**P**]ain) and a diagnostic tool for emergency physicians to focus on specific pathology ([**S**]ize of ovary, [**H**]ydronephrosis, [**A**]ppendicitis, [**R**]ight upper quadrant free fluid, [**P**]regnancy). It is an easily reproducible, cost-effective, and non-invasive study that may expedite risk stratification, diagnosis, and treatment of several emergent conditions.

## Introduction

**Figure 1.**
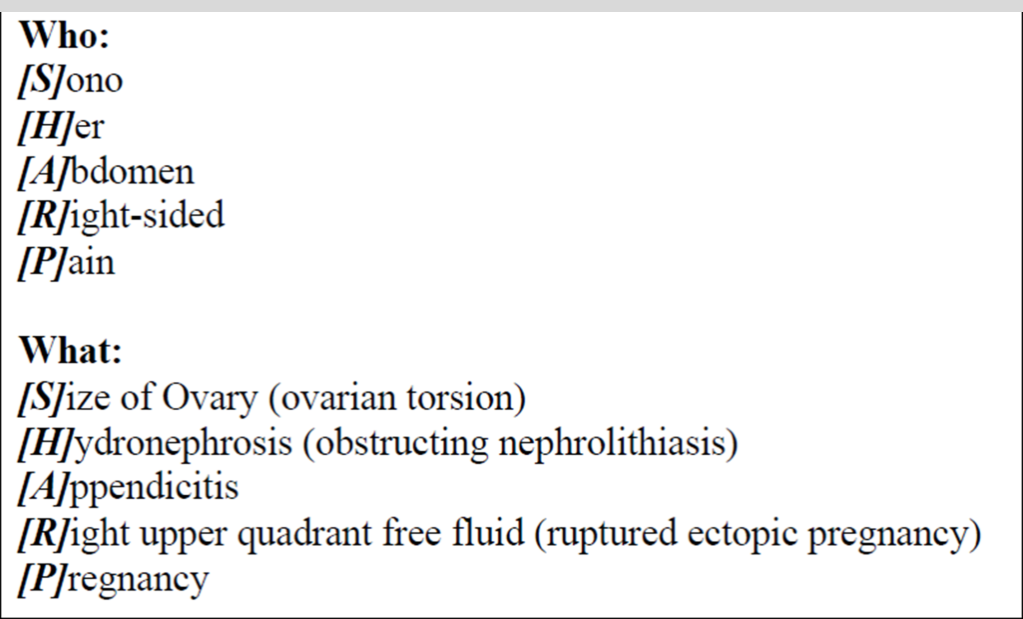
The SHARP Exam nomenclature as an acronym and instruction on key diagnoses.

**Figure 2.**
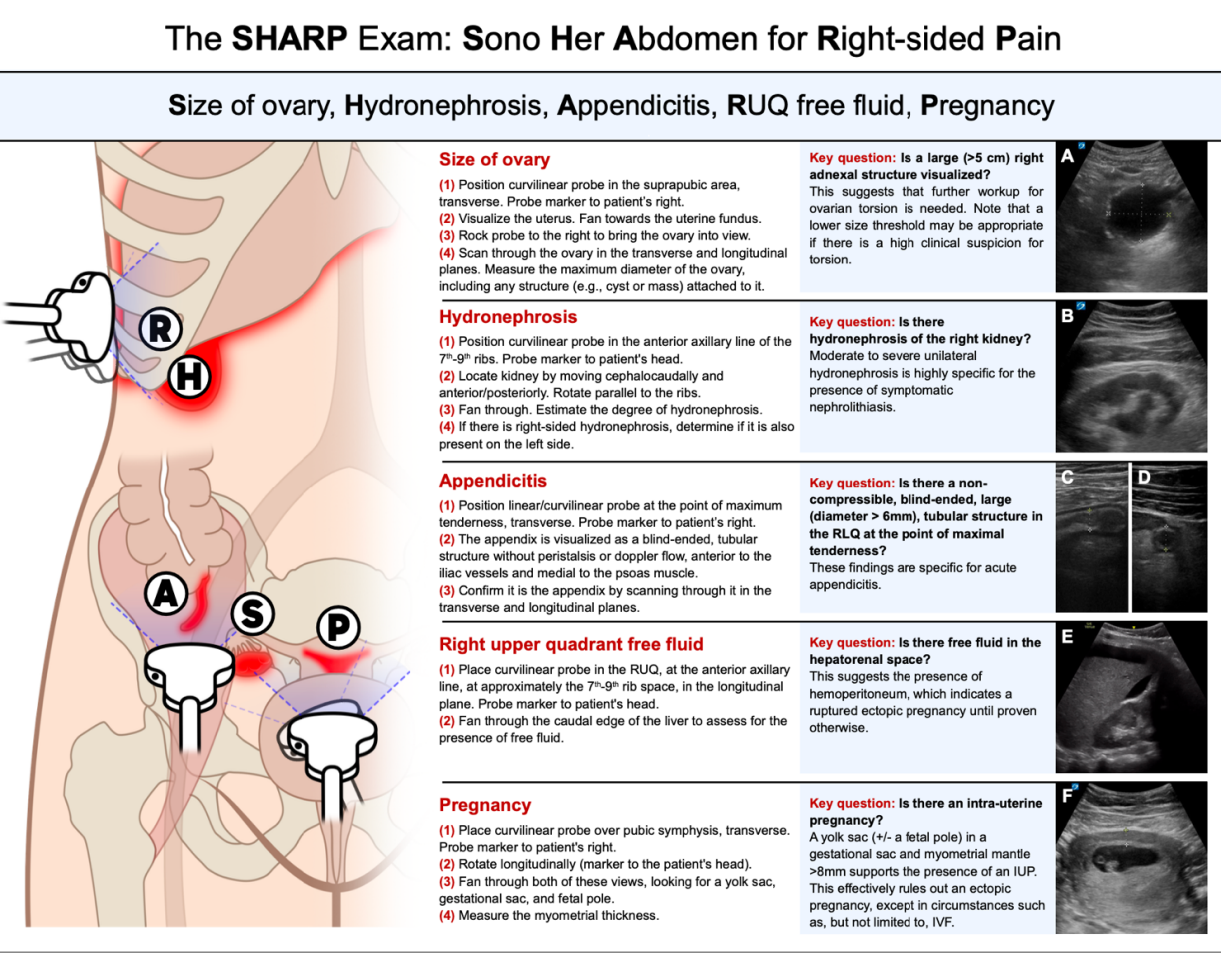
A diagram demonstrating the stages and transducer location of the SHARP protocol.

Abdominal pain is a common presenting complaint in emergency departments. Among those visits, acute right lower quadrant (RLQ) pain in young women of child-bearing age is not infrequent. The differential diagnosis for such a complaint in this demographic conveniently includes several pathologies which can be evaluated quickly with the use of point of care ultrasound (POCUS). The authors have developed a novel systematic protocol using POCUS to rapidly assess for many of these diagnoses, called the SHARP Exam. SHARP is an acronym which defines the indication for the exam (“**S**ono **H**er **A**bdomen for **R**ight lower quadrant abdominal **P**ain”) and also one which guides clinicians to systematically evaluate diagnoses which must not be missed: Size of ovary (torsion), Hydronephrosis (nephrolithiasis), Appendicitis, Right upper quadrant free fluid (ruptured ectopic), and Pregnancy (Figure 1). While the acronym is meant to remind clinicians of the potential pathology involved, the sequence of the SHARP Exam should be informed by the clinician's pretest probability of disease (Figure 2).

The SHARP Exam can be used in any practice setting with access to POCUS. Utilizing this exam, clinicians can stratify patients by risk and potentially arrive at an accurate diagnosis more quickly. Emergency physicians have become quite comfortable with core ultrasound exams and protocols pertaining to particular patient presentations, such as the Extended Focused Assessment of Sonography for Trauma (eFAST) and the Rapid Ultrasound in Shock and Hypotension (RUSH) exams [1–4]. The authors recommend the SHARP Exam be considered in any young woman with undifferentiated RLQ abdominal pain.

## The SHARP Exam: What Clinicians Should Look for on POCUS.

***#1 [S]ize of Ovary*: Is a large (>5 cm) right adnexal structure visualized, yes or no?** *Visualization of a large, right-sided adnexal structure (including an enlarged ovary, ovarian mass, or ovarian cyst) is suggestive of ovarian torsion and can expedite gynecological consultation [5,6].*

**Figure 3.**
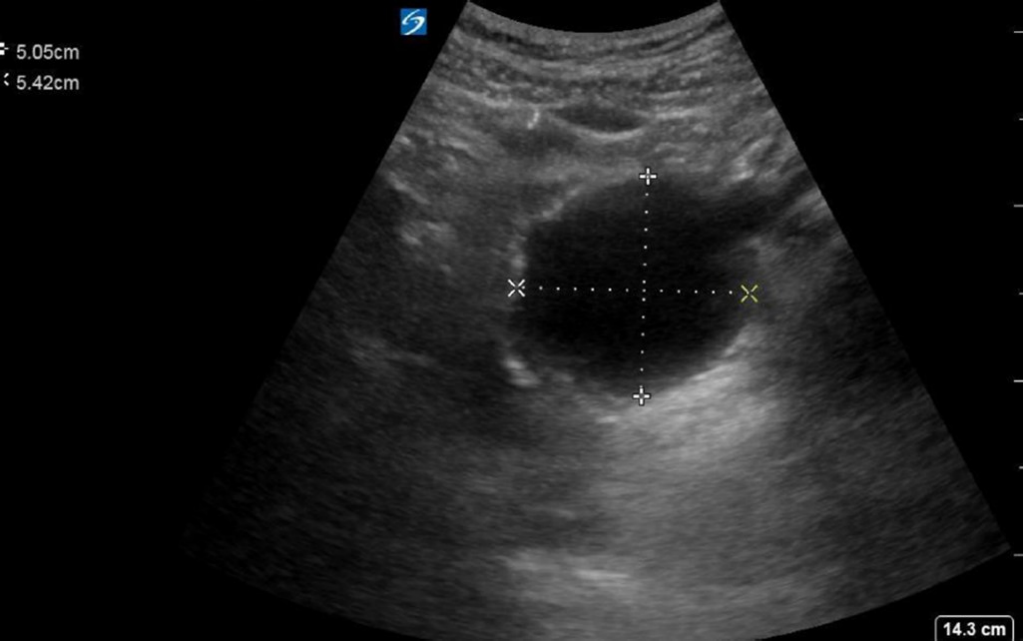
Point of care ultrasound (POCUS) demonstrating diameter measurements for an ovarian cyst (5.05 x 5.42 cm).

Ovarian torsion involves total or partial rotation of the ovary upon its vascular axis. This can prevent flow and lead to thrombophlebitis, loss of function, and eventual ischemia [6–9]. While any woman, regardless of her age, can suffer from ovarian torsion, it is most commonly seen in younger patients with the following risk factors: organically enlarged ovaries (>20 cm3 in premenopausal women, >10 cm3 in postmenopausal women), large ovarian cysts or masses (>5 cm diameter (Figure 3), and patients with ovarian hyperstimulation [8,9,11]. It is a time-sensitive gynecologic emergency that places patients at risk for necrosis and subsequent loss of the affected ovary, peritonitis, and death [6–9]. This diagnosis should be considered in any woman presenting with sudden-onset, unilateral, lower abdominal pain. Emergency physicians should aim to limit any diagnostic delays [8,9]. When ovarian torsion is clinically suspected, POCUS can be performed to visualize the ovaries and expedite the diagnosis.

**Transabdominal Scanning Technique:** To visualize the ovaries utilizing transabdominal POCUS, the clinician should initially locate the uterus with the curvilinear transducer. They should then scan laterally to the sides of the uterus, using the iliac vessels as landmarks and angling the transducer superiorly to identify the ovaries. The ovaries will appear as ovoid structures posteriorly and laterally to the uterus. A full bladder functions as an acoustic window and may improve visualization. Transverse and longitudinal planes should be utilized. The disadvantage of the transabdominal approach is that the pelvic organs are several centimeters away from the abdominal wall. Thus, the lower frequency curvilinear transducer can limit the finer details necessary to identify torsion.

**Transvaginal Scanning Technique:** When the suspicion for torsion is high, the endocavitary transducer should be utilized without delay. It has better resolution and can allow for easier identification of the ovaries. To visualize the ovaries utilizing transvaginal POCUS, the bladder should be empty. A full bladder can interfere with the ability to properly view the uterus and surrounding structures. The endocavitary transducer should be oriented with the indicator initially facing towards the ceiling. The endometrial stripe can be visualized in a midline sagittal plane of view. The uterus should be scanned in both sagittal and coronal planes by rotating the transducer 90 degrees. To identify the ovaries, the transducer can be adjusted to sit against the adnexa. The ovaries will appear ovoid shaped with hypoechoic follicles, medial to the iliac vessels and lateral to the cornual region of the uterus.

While the definitive diagnosis of ovarian torsion is made surgically, POCUS is intended to aid in risk stratification. Specific POCUS findings may guide a clinician to pursue further management and workup for the disease. In a cohort of 92 surgically confirmed cases of ovarian torsion, a maximum ovarian diameter exceeding 5 cm demonstrated a specificity of 91% and sensitivity of 92%, with a positive likelihood ratio of 11.2 and a negative likelihood ratio of 0.09 [12]. Other highly specific ultrasound findings suggestive of ovarian torsion include ovarian edema (visualized as peripherally displaced follicles), the “whirlpool sign,” free fluid in the pelvis, and decreased or absent venous or arterial Doppler flow [8]. Such findings should prompt the emergency physician to obtain an emergent consultation to obstetrics and gynecology for a discussion of the POCUS findings. Though the maximum ovarian diameter is the main indicator for risk of torsion, many emergency physicians may not feel confident identifying or measuring the ovarian diameter on transabdominal POCUS. Most emergency physicians, however, could identify a large mass or cystic structure if present, which would heighten suspicion for torsion. In cases of overt pathology identified with POCUS, the decision can be made to emergently transport the patient to the operating room without further delay [13]. POCUS has been shown to reduce emergency department patient length of stay in non-pregnant patients with gynecologic complaints [14].

***#2 [H]ydronephrosis*: Is there hydronephrosis of the right kidney, yes or no?** *Moderate to severe unilateral hydronephrosis is highly specific for the presence of symptomatic nephrolithiasis [15].*

Hydronephrosis is defined as enlargement of the renal pelvis secondary to obstructive pathology. The layers of the kidney from the periphery to the center include: Gerota's fascia (i.e., the renal capsule), renal cortex, renal medulla (which forms the pyramids), renal calyces, and renal pelvis [16,17]. The renal pelvis is the most echogenic of the layers, appearing brighter than the surrounding kidney structures. When the renal pelvis is dilated, it becomes increasingly hypoechoic/anechoic.

**Figure 4.**
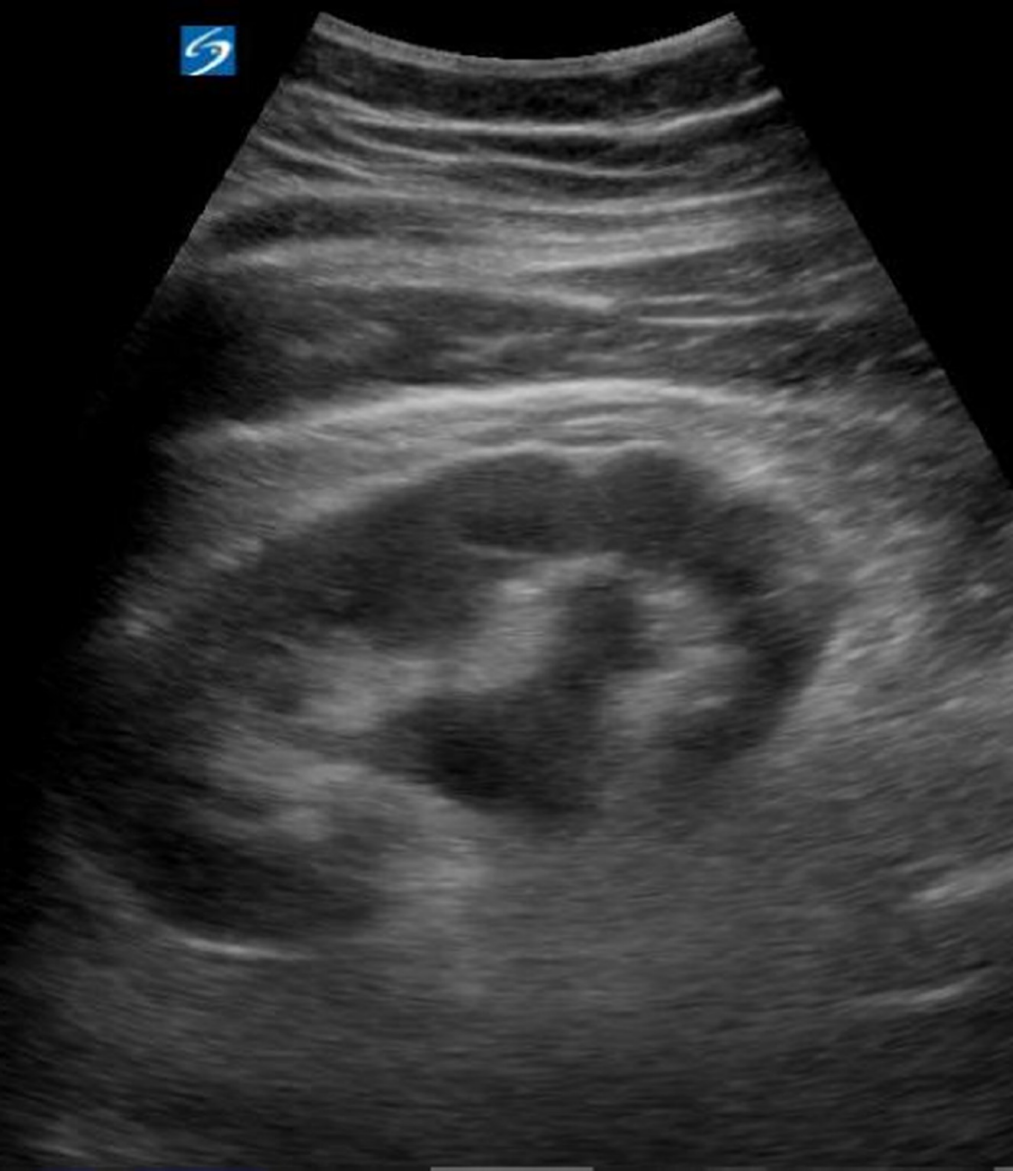
Point of care ultrasound (POCUS) demonstrating hydronephrosis.

**Transabdominal Scanning Technique:** The curvilinear transducer allows for best visualization of the kidneys and can be used to determine the degree of hydronephrosis, if present. The patient should initially be in the supine position. When evaluating the right kidney, the transducer should be placed in the anterior axillary line in the region of the 7th to 9th ribs, with the transducer marker facing towards the patient's head. The transducer can be moved cephalocaudally, as well as anteriorly and posteriorly. The kidneys lie at an oblique angle. In order to avoid visual obstruction by the ribs and to obtain the optimal view, the transducer can be rotated 10 to 20 degrees posteriorly to allow for the transducer to lie parallel to the ribs and allow the beams to pass through without interference. If needed, visualization can be improved by asking the patient to take and hold a deep breath, which lowers the diaphragm and kidney. If there is right-sided hydronephrosis, the left kidney should also be evaluated as bilateral hydronephrosis could lead the clinician down a different diagnostic pathway (Figure 4). When POCUS demonstrates severe hydronephrosis, it may be appropriate to advance to further imaging, such as computed tomography (CT) or medical resonance imaging. In the absence of this situation, POCUS can be used as the sole imaging modality to diagnose nephrolithiasis. No significant difference has been found in “high-risk diagnoses, adverse effects, and repeat emergency department visits between ultrasound and CT [18].”

**Figure 5.**
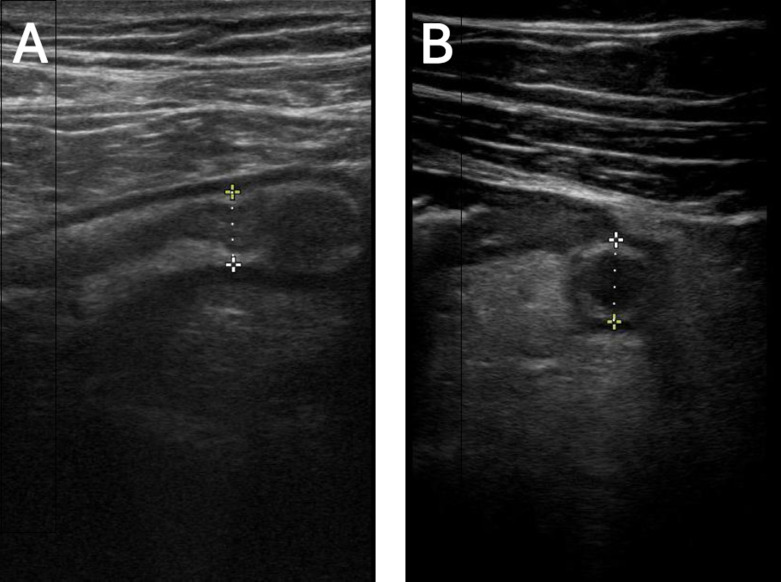
Point of care ultrasound (POCUS) findings with anterior-posterior appendiceal diameter greater than 6mm in longitudinal (A) and transverse (B) views.

***#3 [A]ppendicitis*: Is there a non-compressible, blind-ended, large (diameter >6 mm), tubular structure in the RLQ at the point of maximal tenderness, yes or no?** *These findings are specific for acute appendicitis (Figure 5).*

Acute appendicitis refers to inflammation of the appendix. While the pathophysiology is poorly understood, likely etiologies include infectious agents, genetic factors, or direct luminal obstruction through fecalith, lymphoid hyperplasia, or impacted stool. CT is currently the gold-standard for diagnosis, with 98.3% sensitivity [19–22]. In settings where CT is readily available, the study is most accurately performed with intravenous (IV) contrast. IV contrast can prolong the time to diagnosis, as the protocol in many institutions requires that laboratory testing indicate that renal function have resulted prior to IV contrast administration.

POCUS is readily available and provides an expeditious tool to assess for appendicitis [20,21]. While not traditionally part of core POCUS examinations performed by emergency physicians, many studies support its utilization in this setting. Pooled sensitivity and specificity of emergency physician-performed POCUS for appendicitis have been reported, ranging from 0.81 to 0.92 and 0.63 to 0.96, respectively [23–25]. Positive and negative likelihood ratios have been reported at 2.29 (95% CI: 1.85-2.84) and 0.24 (95% CI: 0.14-0.42), respectively [24]. These studies also demonstrate a positive impact on emergency physician clinical decision making. Furthermore, a systematic review and meta-analysis of 21 studies by Cho and Oh demonstrates the accuracy of POCUS for the diagnosis of acute appendicitis in the emergency department [26]. They reported a pooled sensitivity of 0.81 (95% CI: 0.78-0.83) and specificity of 0.87 (95% CI: 0.85-0.88), with high levels of accuracy (Area Under the Curve 0.9249, Standard Error 0.0180) [26]. However, limitations of POCUS for the diagnosis of appendicitis include high operator dependence, difficulty visualizing the appendix due to patient body habitus, and poor visualization in certain anatomic variations and where there is increased bowel gas.

**Transabdominal Scanning Technique:** Either the linear or curvilinear transducer can be used to evaluate appendicitis. While the linear transducer is generally preferred, the curvilinear transducer may provide better visualization through increased depth in patients with greater body mass indices. The ideal transducer placement for identification of the appendix is on the RLQ, particularly at the point of maximal pain or tenderness to palpation. The transducer should be oriented in the transverse plane and the transducer marker should be pointed to the patient's right shoulder. The anatomic landmarks for identifying the appendix are the psoas muscle and the iliac vessels. The psoas muscle can be seen on the left side of the screen (laterally on the patient) and the iliac vessels will be on the right side of the screen (medially on the patient). The appendix lies anterior to the iliac vessels and will be closer to the top of the screen. The appendix can be differentiated from other structures as it is a blind-ended, non-compressible tubular structure without peristalsis or Doppler color flow.

***#4 [R]ight Upper Quadrant Free Fluid*: Is there free fluid in the hepatorenal space, yes or no?** *This suggests the presence of hemoperitoneum, which indicates a ruptured ectopic pregnancy until proven otherwise. The caudal edge of the liver must be evaluated, as it is the most sensitive area in the assessment of free fluid (hemoperitoneum) [27].*

An ectopic pregnancy is defined as a fertilized egg that implants outside of the uterus, often in the fallopian tubes. Physical exam alone is insufficient to rule out this life-threatening condition and normal vital signs can be falsely reassuring, as they do not correlate with hemoperitoneum [28]. Early POCUS that identifies free fluid in the hepatorenal space is therefore critical, as free fluid has been shown to predict the need for operative intervention [29]. While free fluid does not definitively rule in a ruptured ectopic pregnancy, in the context of a positive pregnancy test and no intrauterine pregnancy, it is the diagnosis of greatest concern.

Use of POCUS for determination of internal bleeding has been well studied in trauma with the eFAST examination. While much interest in the eFAST examination has been focused on trauma, eFAST may also indicate significant non-traumatic pelvic and intraperitoneal hemorrhage [27]. Rodgerson et al. demonstrated that identifying patients with a suspected ectopic pregnancy and fluid in Morison's pouch by emergency physician-performed bedside ultrasound decreased the time to diagnosis and treatment [30]. Furthermore, Moore et al. demonstrated that rapid identification of free intraperitoneal fluid in Morison's pouch in patients with suspected ectopic pregnancy by emergency physician-performed POCUS correlated with the need for operative management [29]. Free fluid in Morison's pouch was identified in 10 patients, 9 of whom underwent operative intervention, yielding a positive likelihood ratio of 112 (95% CI: 15-831). The high likelihood ratio of free fluid for operative intervention suggests that the addition of a Morison's pouch view may provide immediate and useful information regarding patient disposition.

**Figure 6.**
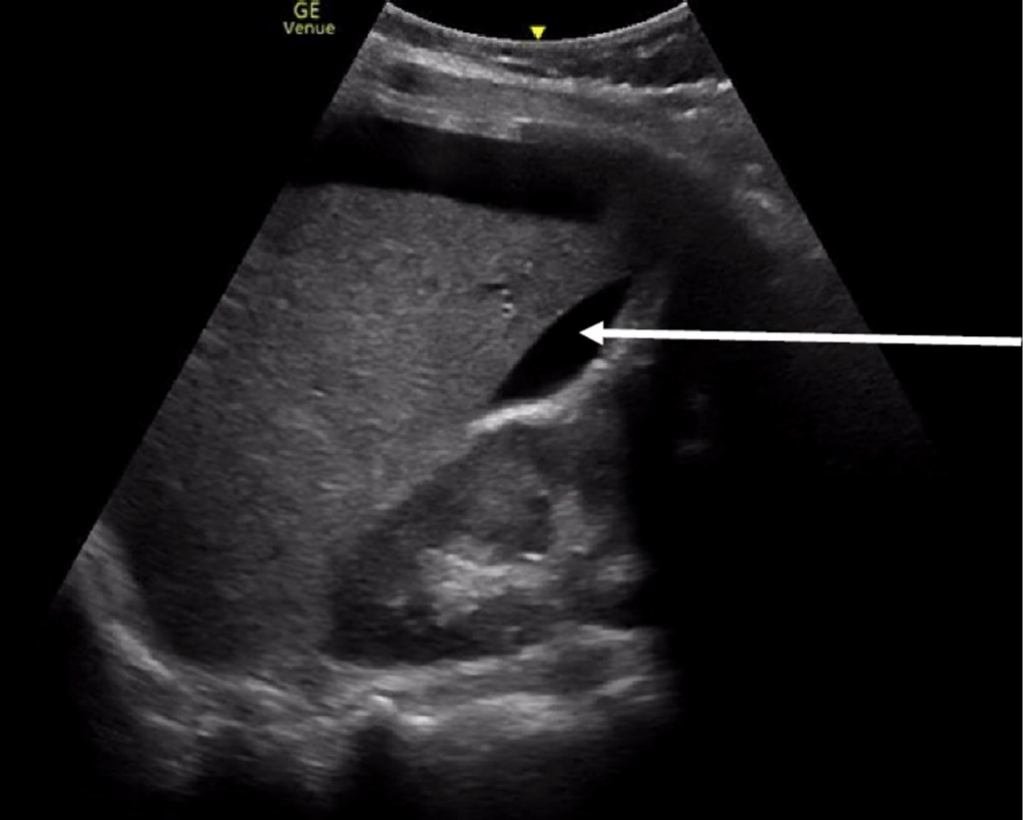
Point of care ultrasound (POCUS) demonstrating anechoic free fluid in the right upper quadrant along the hepatorenal space (white arrow) consistent with a ruptured ectopic pregnancy.

**Transabdominal Scanning Technique:** For a patient with a positive pregnancy test and no definitive intrauterine pregnancy observed on POCUS, a comprehensive view of the right upper quadrant should always be obtained by the emergency physician. The presence of intra-abdominal free fluid should raise concern for ruptured ectopic pregnancy. This examination is performed using standard ultrasound equipment present in many emergency departments today. Many authors recommend using a low-frequency (3.5 MHz) transducer, such as the curvilinear array, as this provides excellent intra-abdominal views. With the patient in a supine position and the transducer maker pointing cranially, the starting position for the right upper quadrant view should be the anterior axillary line, at approximately the 7th to 9th rib space. Fanning through the area can help obtain a comprehensive view of the right upper quadrant. This ensures visualization of the caudal edge of the liver which is the most sensitive area in the assessment of free fluid (Figure 6).

***#5 [P]regnancy*: Is there an intrauterine pregnancy? Yes or no.** *A yolk sac (+/- a fetal pole) in a gestational sac and myometrial mantle >8 mm supports the presence of an intrauterine pregnancy. This effectively rules out an ectopic pregnancy, except in exceedingly rare circumstances such as, but not limited to, heterotopic pregnancies.*

Evaluation of the uterus seeks to confirm an intrauterine pregnancy, which in most situations effectively rules out an ectopic pregnancy. Ectopic pregnancies can occur with any human chorionic gonadotropin (hCG) level and have been described with levels <30 IU/mL [31,32]. As such, pelvic POCUS should be done for any patient who is pregnant with concerning symptoms regardless of hCG level. The discriminatory zone is the level of beta-hCG at which an intrauterine pregnancy should be visualized 100% of the time. For transabdominal ultrasound, it is 4000-6600 IU/mL. Thus, if a POCUS is indeterminate and the hCG level is above the discriminatory zone, the suspicion for ectopic pregnancy should be increased.

**Figure 7.**
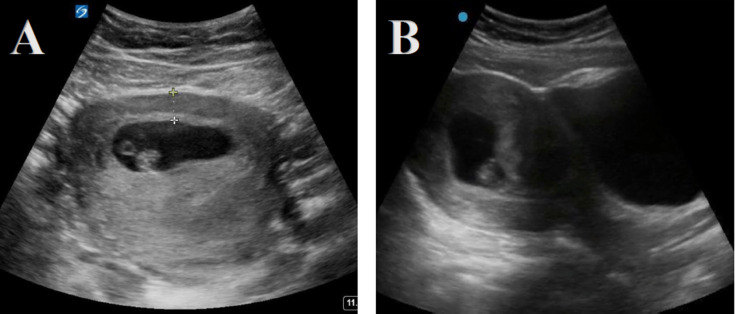
Point of care ultrasound (POCUS) demonstrating an intrauterine pregnancy (a gestational sac containing a yolk sac within the endometrium in 2 planes) with measurement of the myometrial mantle.

The focused question for the first trimester POCUS is whether there is an intrauterine pregnancy present. An intrauterine pregnancy is defined as a gestational sac containing a yolk sac within the endometrium in two planes [33] (Figure 7). It is important to ensure that at least 8 mm of endometrium is surrounding the gestational sac so as to not miss an interstitial ectopic pregnancy. This measurement is termed the “myometrial mantle.” A gestational sac alone is not sufficient to determine an intrauterine pregnancy. An estimated10-20% of ectopic pregnancies have a pseudo-gestational sac [33]. A visualized intrauterine pregnancy on POCUS effectively rules out ectopic pregnancy, excluding specific circumstances such as in-vitro fertilization in which the likelihood of a heterotopic pregnancy significantly increases. The pooled sensitivity of emergency physician-performed evaluation for intrauterine pregnancy is 99.3% (95% CI: 96.6-100%) [27]. This data comes from studies that included both transvaginal and transabdominal pelvic POCUS examinations, so if there is concern for ectopic pregnancy and no intrauterine pregnancy is seen on transabdominal POCUS, the patient requires a transvaginal ultrasound to further evaluate.

**Transabdominal Scanning Technique:** The preferred transducer to evaluate for an intrauterine pregnancy via the transabdominal approach is the 3.5-5 MHz curvilinear transducer. The patient should be in supine position. The transabdominal assessment is best obtained with a full urinary bladder, which is an acoustic window to identify the uterus. An inadequately filled bladder is one of the most common reasons for a technically inadequate transabdominal examination. The uterus is a globe-shaped structure, so at least two views are necessary for complete evaluation of the uterus: transabdominal and longitudinal views. To begin, the transducer should be placed over the pubic symphysis with the indicator pointed to the patient's right to obtain the transverse view of the uterus. Next, fan all the way through by angling the transducer towards the head and moving downward towards the feet. To obtain the sagittal plane, rotate the transducer towards the operator's right-side, with the indicator pointing to the patient's head, then fan left to right to fan through and obtain images.

**Transvaginal Scanning Technique:** The preferred transducer to evaluate for an intrauterine pregnancy via the transvaginal approach is the high-frequency intracavitary transducer (ranging from 5-9 MHz). After performing the transabdominal ultrasound, the patient should empty their bladder. The patient should be supine in a lithotomy position. Using a sterile transducer, the transducer tip should be covered with gel, then a sterile cover should be placed over the transducer with a small amount of sterile conducting gel. Two views are necessary for complete evaluation of the uterus: one transverse view and one longitudinal view, both demonstrating the endometrial stripe and uterine contents, if present. With the transducer held with the indicator pointed toward the ceiling, the transducer should be inserted into the vaginal canal. In the sagittal plane, the transducer should be moved side to side, fanning through the entire body of the uterus. Angling the transducer handle toward the ceiling will bring the cervix into view (angling the tip toward the posterior fornix of the vaginal canal). Next, the transducer should be rotated toward the patient's right to obtain the transverse view. Fanning the transducer up and down will show anterior to posterior views of the uterus. From this point, the fallopian tubes can be followed to visualize the ovaries. If the iliac vessels can be observed, the ovaries should be anterior and medial to the vessels. The ovaries can be identified by the multiple follicles.

## Conclusion

Acute RLQ abdominal pain in young female patients of child-bearing age is a common presentation in the emergency department. The workup of such patients varies widely depending on the clinical setting. The SHARP Exam was designed as a novel POCUS protocol for evaluating this patient population. The SHARP Exam may make a difference in the rapid diagnosis at the point of care of intrauterine pregnancy, ruptured ectopic pregnancy, obstructing nephrolithiasis, appendicitis, and ovarian torsion. The SHARP Exam is akin to the eFAST and RUSH exams that are applied to specific patient presentations, trauma and hypotension of medical etiology respectively. It offers a framework for systematically evaluating for dangerous pathology in a specific patient population—women of child-bearing age with undifferentiated RLQ abdominal pain. The authors believe that integrating the SHARP Exam may aid in the care of this vulnerable patient population.
